# Efficacy and Safety of Dahuang Zhechong Pill in Silicosis: A Randomized Controlled Trial

**DOI:** 10.1155/2021/4354054

**Published:** 2021-11-18

**Authors:** Wu-Yi-Nuo Tang, Jing-Tao Liang, Ju Wu, Li Liu, Ming-Zhang Lu, Xiao-Yan He, Li-Juan Wu, Huan-Yu Jiang, Fei Wang, Xiao Meng, Shun-Pin Li

**Affiliations:** ^1^Chengdu University of Traditional Chinese Medicine, Chengdu 610075, China; ^2^Panzhihua Second People's Hospital, Panzhihua City, Sichuan Province, China

## Abstract

**Background:**

There is no effective therapy for silicosis, and Dahuang Zhechong pill (DHZCP), an ancient Chinese medicine prescription, may have a therapeutic effect on silicosis. This study aims to verify the efficacy and safety of DHZCP in silicosis.

**Methods:**

This is a randomized controlled clinical trial done at Panzhihua Second People's Hospital (Panzhihua City, Sichuan Province, China). Participants diagnosed with silicosis were recruited and randomized to the conventional treatment group (CG) or DHZCP combined with the conventional treatment group (DG). Forced vital capacity % predicted (FVC%), diffusing capacity of the lung for carbon monoxide % predicted (DLCO%), six-minute walk distance (6MWD), peripheral oxygen (SpO_2_), King's Brief Interstitial Lung Disease Questionnaire (K-BILD), and safety outcomes were measured at baseline and 9 weeks.

**Results:**

Fifty-six participants (28 in each group) completed the study, and 53 of them (26 in DG and 27 in CG) completed pulmonary function. At 9 weeks, compared with no DHZCP, DHZCP treatment was associated with significant improvements in FVC% (mean ± SD, 95%CI) (8.2 ± 3.9, 0.3 to 16.0), DLCO% (8.6 ± 3.5, 1.5 to 15.7), SpO_2_ (3.8 ± 0.7, 2.3 to 5.2), and K-BILD total score (6.0 ± 2.3, 1.4 to 10.7). And, there were no statistical differences of safety outcomes between the two groups. Eight patients accepting DHZCP developed mild diarrhea during the first week, which subsequently resolved on its own.

**Conclusion:**

DHZCP could improve the pulmonary function, the quality of life, and the exercise capacity of silicosis patients.

## 1. Introduction

Silicosis is a fatal and irreversible fibrotic pulmonary disease, which is one of the most common occupational respiratory diseases worldwide [1, 2]. It is caused by the inhalation and deposition of respirable crystalline silica (SiO_2_) (particles <10 *μ*m in diameter), so silica dust plays a key role in pulmonary fibrosis and inflammation [3, 4]. Silica particles are inhaled and then reach the lower respiratory tract and the gaseous exchange zones. Alveolar macrophages will phagocytose them, and they can cause an inflammatory process that is characterized by the production of reactive oxygen species (ROS). Eventually, ROS will damage the pulmonary parenchyma, and the subsequent repair/regeneration process result in fibrogenesis and carcinogenesis [1, 5, 6]. The manifestations of silicosis include, but not limited to, shortness of breath at rest or on exertion, reduced exercise capacity, and health-related quality of life (HRQoL) [7–9]. Although there have been many studies, the pathogenesis of silicosis is not completely clear; therefore, the deterioration of silicosis can only be slowed down by comprehensive management, but there is no real effective therapy [1, 10].

Traditional Chinese Medicine (TCM) is not the mainstream therapy for silicosis, while it has been increasingly adopted as a way of complementary and alternative medicine worldwide [11]. Chinese herb formulas, although they have not been accepted, are the most commonly used TCM treatment in China. A lot of studies have reported that TCM interventions have positive significance for improvement of HRQoL and reduction of symptoms of pulmonary fibrosis patients [12]. In recent years, there have been more and more systematic reviews and meta-analyses about application of TCM in pulmonary fibrosis [13–17].

Pulmonary fibrosis belongs to “the atrophic lung disease” in TCM, and the main pathogenesis is blood stasis and deficiency of both “qi” and “yin.” Dahuang Zhechong Pills (DHZCP), a canonical traditional Chinese medicine from “Treatise on Cold Pathogenic and Miscellaneous Diseases (Shanghan Zabing Lun),” which consist of大黄 (Rheum palmatum L.), 黄芩 (*Scutellaria baicalensis* Georgi), 甘草 (Glycyrrhiza uralensis Fisch), 桃仁 (Persicae Semen), 杏仁 (Semen Armeniacae Amarum), 芍藥 (Paeonia lactiflora Pall.), 幹地黃 (*Rehmannia glutinosa* (Gaertn.) Libosch.), 幹漆 (*Toxicodendron vernicifluum*), 虻蟲 (*Tabanus*), 水蛭 (Whitmania Pigra Whitman), 蠐螬 (*Holotrichia diomphalia* Bates), and 䗪蟲 (*Eupolyphaga*), are officially recorded in Chinese Pharmacopoeia. In recent years, some researchers have found that DHZCP has the effect of alleviating Silica-induced pulmonary fibrosis [18]. Therefore, we plan to evaluate the efficacy and safety of DHZCP through this randomized controlled trial and provide an effective pharmacological therapy for silicosis.

## 2. Methods

### 2.1. Sample Size Calculation and Random Grouping

Some studies have reported that diffusing capacity of the lung for carbon monoxide % predicted (DLCO%) of patients with silicosis was (mean ± SD) 59.9% ± 5.9% after conventional treatment, and it was 65.9% ± 6.1% after TCM combined with conventional treatment [19]. Therefore, we assumed that mean DLCO% was 65.9% after DHZCP combined with conventional treatment, and SD was 6.0%. The power of test was 0.9, and alpha was 0.5. According to the proportion of 1 : 1, this study needed at least 23 cases in every group. And dropout rate was considered as 20%, and this study needed 58 cases in total. The sample size was calculated by PASS 15.0 software (Two-Sample T-Test Assuming Equal Variance).

In order to make sure that the sample sizes were equal, block randomization was adopted in the study. The size of each block was set as 4. The group of DHZCP combined with conventional treatment (DG) was set as group A, and the group of no DHZCP but conventional treatment (CG) was set as group B. Then, there were 6 arrangement methods in each block: AABB, ABAB, ABBA, BAAB, BABA, and BBAA. The 6 arrangement methods were numbered from 1 to 6. Generate a string of random numbers with Microsoft Excel 2019. Remove the numbers 7 to 9 from the string of random numbers, and then get a new string of numbers. And press this number to arrange the corresponding number of blocks, obtain a randomized string, and then match the string to the list of patients. The randomization was completed. The generation of the random allocation sequence, the enrollment of participants, and the assignment of interventions were the responsibility of different people.

### 2.2. Participants

The study was conducted in Panzhihua Second People's Hospital (Panzhihua City, Sichuan Province, China) during December 2020 and March 2021. Eligible participants were diagnosed with silicosis. In addition, participants were aged 18 years or older and 85 years or younger and volunteered for this study.

Patients were excluded from the study if they are hypersensitive or allergic to DHZCP; with high risk of bleeding or blood coagulation problems, or any severe bleeding event within 3 months prior to baseline; with cardiac or cerebrovascular accidents or surgeries with a possible secondary bleeding event occurred within 12 months prior to baseline; with the cardiovascular, cerebrovascular, liver, kidney, hematopoietic system, or other serious primary diseases, or abnormal liver or kidney function; pregnant, lactating women; unwilling to accept the study measures; with cognitive dysfunction, mental disorder, or psychosis; drug or alcohol abuse or dependence; participants who had participated in or were participating in any clinical trial of another drug within one month prior to baseline.

### 2.3. Diagnostic Criteria

Diagnosis had been established by the participant's respiratory physician with reference to *Diagnosis of Occupational Pneumoconiosis* (GBZ 70-2015) [20], a standard published by National Health and Family Planning Commission of the People's Republic of China. Silicosis can be diagnosed in patients with a reliable history of exposure to productive mineral dust and confirmed by pulmonary function and X-ray examination.

### 2.4. Intervention

The participants assigned to conventional treatment accepted antiasthmatics (Doxofylline for Injection, Shanxi Bosen Bio-Pharmaceutical Group Co. Ltd, batch number: H20052406), expectorants (Bromhexine Hydrochloride for Injection, Heilongjiang Aolidanaide Pharmaceutical Co. Ltd, batch number: H20100178), antitussive (Feilikeheji mixture, Guizhou Jianxing Pharmaceutical Co. Ltd, batch number: Z20025136) and oxygen therapy (oxygen inhalation through nasal catheterization), and so on [21]. The other participants were additional to accept DHZCP (Beijing Tong Ren Tang Co., Ltd., Z11020002), 6 g twice a day.

All participants were allowed to maintain the original treatments of other diseases, including hypertension and diabetes. All of the original treatments should be regularly used for 3 months prior to baseline. During the study and observation, the type and dosage of the drug should be kept as consistent as possible. If there is any modification, the reason should be explained. And any combined treatment must be recorded with name, dosage, frequency, and duration of use on the case report form for summary analysis and reporting. But other than what is prescribed in the study, treatments for silicosis were not allowed to be used. When patients suffer from septic shock, respiratory failure, and other serious complications, reasonable and standard first aid measures should be taken as soon as possible. If it is necessary, patients can be off the trial.

### 2.5. Study Design and Ethical Considerations

The study was a paralleled, randomized controlled trial. After recruitment into the study and collection of baseline measures, participants were randomized to DG or CG and received the corresponding interventions. And, all of the outcomes were measured at baseline and 9 weeks.

The study was approved by the Research Ethics Committee of Hospital of Chengdu University of Traditional Chinese Medicine. Prior to conducting the trial, written informed consent was obtained from each patient willing to participate after being given a detailed explanation about the trial.

### 2.6. Outcomes

The main manifestation of silicosis is restricted ventilation dysfunction accompanied by decreased pulmonary diffusion function, so the primary outcome was pulmonary function. Forced vital capacity %predicted (FVC%) and diffusing capacity of the lung for carbon monoxide % predicted (DLCO%) were mainly focused on. And pulmonary function was measured with pulmonary function test system (product model: MasterScreen, manufacturer: CareFusion Germany 234 GmbH).

There were some researchers who proved that six-minute walk distance (6MWD) outweighs other predictors of mortality in idiopathic pulmonary fibrosis (IPF) [22]. Since both IPF and silicosis are interstitial lung disease (ILD), and their pathologic changes and clinical manifestations are similar, we chose 6MWD as an index to assess the efficacy of DHZCP. In addition, saturation of peripheral oxygen (SpO_2_) was measured with Finger clip pulse oximeter (product model: YX303, manufacturer: Jiangsu Yuyue Medical Equipment and Supply Co., Ltd.). These two indexes can well reflect the exercise ability and the degree of hypoxia of patients with silicosis.

There were multiple studies that proved that King's Brief Interstitial Lung Disease Questionnaire (K-BILD) [23] can effectively assess the HRQoL of patients with ILD [24–26]. Compared with the modified version of Saint Geoge Respiration Questionnaire for patients with IPF (SGRQ-I), K-BILD has similar validity in the assessment of patients with ILD, but K-BILD is more concise. Considering the evaluation efficiency, we finally adopted K-BILD. K-BILD is composed of 15 questions, each with a seven-point response scale, which are grouped into three domains: breathlessness and activities, chest symptoms, and psychological symptoms. The individual domain and total scores range from 0 to 100, with lower scores indicating worse quality of life.

Safety outcomes were red blood cell (RBC), hemoglobin (HGB), alanine transaminase (ALT), aspartate transferase (AST), blood urea nitrogen (BUN), serum creatinine (SCr), myohemoglobin (Myo), cardiac troponin I (cTnI), prothrombin time (PT), international normalized ratio (INR), and activated partial thromboplastin time (APTT). Because of the composition of DHZCP, particular attention should be paid to coagulation function and liver and kidney function. All of these outcomes were measured at clinical laboratory of Panzhihua Second People's Hospital.

### 2.7. Statistical Analysis

All statistical analyses were performed with the Statistical Package for Social Sciences (SPSS) software, version 22 (IBM, USA). Categorical variables are reported as numbers and percentages, while continuous variables are reported as means ± standard deviations (SD). Statistical differences between groups were determined by independent-samples *t*-test for continuous variables and by the Pearson chi-square test for binary categories variables. Statistical differences within groups were determined by paired-sample *t* test. A *p* value of <0.05 was considered the cutoff value of statistical significance. Data missing at either baseline or 9 weeks was excluded from the analyses.

## 3. Results

**Figure 1:**
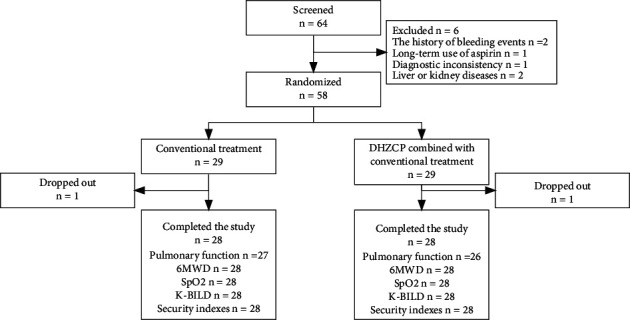
Flow of participants through the study.

Between December 2020 and March 2011, 64 participants were screened, with 58 being randomized. The reasons for noninclusion of six participants were the history of bleeding events within three months (two), long-term use of aspirin (one), diagnostic inconsistency (one), and liver or kidney diseases (two). The flow of participants through the study is shown in Figure 1. All of 58 participants were male, and their baseline data were recorded in detail. Baseline characteristics of participants are shown in Table 1.

25 (86.2%) participants of DG had smoke history with an average age of (mean ± SD) 71.8 ± 9.0 years, and 24 (82.8%) participants of CG had smoke history with an average age of (mean ± SD) 69.3 ± 8.0 years. There were no significant differences between DG and CG (*p* > 0.05) regarding general information, pulmonary function, hypoxia and exercise ability, and K-BILD score. The comparison of their baseline characters is shown in Table 1.

At nine weeks, 56 participants completed the study, and 3 patients (2 of DG and 1 of CG) did not take pulmonary function tests for personal reasons, but they completed other programmes. Two patients (1 of DG and 1 of CG) without taking the medicine as prescribed were excluded, and after excluding their data, participants on the two interventions remained comparable at baseline (*p* value >0.05 when all information for both interventions was compared). All of 56 patients were treated exactly as prescribed in the study.

The effects of different treatments at 9 weeks are shown in Table 2, and they are compared in Table 3. DG showed a greater improvement in pulmonary function than CG. Comparing DG to CG, the mean difference (MD) of FVC% was 8.15, the standard error (SE) was 3.92, the confidence interval (CI) was 0.28 to 16.02, and the *p* value was 0.043. The MD of DLCO% was 8.64, the SE was 3.53, the CI was 1.555 to 15.73, and the *p* value was 0.018.

The improvement of hypoxia in DG was better. The MD of SpO_2_ was 3.79, the SE was 0.72, the CI was 2.34 to 5.23, and the *p* value <0.001. However, there was no significant difference of 6MWD between two groups.

The HRQoL of DG was also better than CG. The MD of K-BILD total score was 6.03, the SE was 2.33, the CI was 3.22 to 18.20, and the *p* value was 0.012. And there were also significant differences of the other parts of K-BILD between the two groups.

Statistical differences of the two treatments between after and before intervention are shown in Table 4. DG had a statistically significant difference in the 6MWD test (39.64 min, 28.06–51.23) in week 9 compared to baseline, as well as FVC% (10.37%, 6.63–14.11), DLCO% (10.51%, 6.73–14.29), SpO_2_ (3.61%, 2.86–4.36), breathlessness and activities (8.48, 5.75–11.21), K-BILD Chest symptoms (11.31, 8.41–14.21), and K-BILD psychological (2.72, 1.55–3.89).

None of the participants showed any change from normal to abnormal safety outcomes before and after intervention (Table 5). But there were 8 patients of DG who developed mild diarrhea during the first week of taking the drug. They had diarrhea for an average of 4.6 days and defecated 2.4 times a day on average. The diarrhea disappeared spontaneously within a week; none of them used antidiarrheic (Table 6).

## 4. Discussion

To the best of our knowledge, this is the first randomized controlled trial to assess the efficacy and safety of DHZCP in silicosis. This study demonstrated that the combination of DHZCP and conventional treatment has an advantage over conventional treatment in improving pulmonary function and HRQoL in patients with silicosis. Mild diarrhea in participants disappeared spontaneously, and further studies are needed to confirm the safety of DHZCP for patients with silicosis.

DHZCP improved pulmonary function measured by an increase of FVC (10.37%) and DLCO (10.51) in this cohort of patients with silicosis. These changes in pulmonary function indicate not only improvement in the patients' restricted ventilation dysfunction, but also improvement in pulmonary diffusion function. This proves that DHZCP can effectively improve the pulmonary dysfunction caused by silicosis.

It also improved exercise capacity by an increase of 39.64 meters in 6MWD. Although there were some researchers who verified the effects of exercise training in asbestos-related and other dust-related respiratory diseases [27], evidence that exercise training improves pulmonary function in patients with silicosis remains lacking. Compared to the study by Dale et al., the improvement of 6MWD in our patients seemed to be less significant [27]. But considering the race, disease range, baseline conditions, and other factors, it cannot be concluded that DHZCP is inferior to exercise in the treatment of silicosis. And the improvement of DHZCP on hypoxia is also very significant, which can be proved by the elevation of SpO_2_.

Moreover, since silicosis is a refractory and fatal disease, it is important for patients with silicosis to live longer and improve their HRQoL. DHZCP is effective in improving HRQoL of patients with silicosis, with the improvement of K-BILD total (5.79), breathlessness and activities (8.48), chest symptoms (11.31), and psychological symptoms (2.72).

There have been few studies on drug treatment of silicosis, so it is difficult to find a comparison with this study. Therefore, according to the existing studies, we can conclude that DHZCP has unique advantages in the treatment of silicosis.

From the perspective of modern pharmacological studies, some major components of DHZCP are helpful in improving pulmonary fibrosis. Rheum palmatum can exert bacteriostatic, anti-inflammatory, and antiviral action through emodin [28]. So, it can reduce the incidence of pulmonary infections and inflammation in the lungs. Some studies have reported that rheum officinale can effectively reduce airway inflammation in mice [29]. *Eupolyphaga* was confirmed to contain a kind of protein with both direct-acting fibrinolytic and plasminogen-activating activities [30, 31]. It also has a lipid-lowering effect [32], and some researchers have found that lipid metabolism is closely related to pulmonary fibrosis [33–35]. The active ingredients and effects of all components of DHZCP are shown in Table 7.

Persistent inflammatory stimulation is a crucial cause of fibrosis. Inhalation of SiO_2_ causes persistent pulmonary inflammation, leading to the deposition of the extracellular matrix, and ultimately to the fibrosis of lung tissue [36]. Our team has carried out some studies about the mechanism of DHZCP against fibrosis. Various components of DHZCP can reduce fibrosis by anti-inflammatory, which has been confirmed to be related to the effects of DHZCP in inhibiting of NF-*κ*B p65 and the blocking of TGF-*β*1/Smad pathway and downregulating the expression of p38 MAPK and p-p38 [18, 37].

Transforming growth factor-*β*1 (TGF-*β*1) is a kind of multifunctional cytokine with a wide range of biological activities. It plays a fundamental role in the regulation of cell processes, such as cell growth, apoptosis, and differentiation, and is widely recognized as a promoter of the formation and development of pulmonary fibrosis [38–44]. There was a study reporting that the concentration of TGF-*β*1 in the silicified rat models is significantly higher than that in the saline control group [10].

Moreover, multiple studies have reported that DHZCP can slow down or even reverse the progression of fibrosis by decreasing the levels of collagen and inactivating the PI3K/Akt pathway [45, 46]. These studies have certain reference value, though they are mostly focused on hepatic fibrosis. Therefore, it is valuable to further study the relationship between DHZCP and the TGF-*β*1/Smad pathway. And because of the antifibrotic effects of DHZCP, it may also be effective in other fibrotic diseases, such as idiopathic pulmonary fibrosis.

There were 8 patients of DG developed mild diarrhea, and it may be related to the laxative effect of some drugs in DHZCP. Rhein and emodin in *Rheum palmatum* L. have significant laxative effects. Persicae Semen and Semen Armeniacae Amarum contain a lot of lipids, which can lubricate intestinal tract. And all of the patients with diarrhea became healthy within a week, which demonstrated that DHZCP is safe for patients with silicosis.

Our study provides helpful insights into the clinical improvement of silicosis, but there are still some limitations. Firstly, study population of our trial is small. All of the participants were male. Secondly, due to the limitation of hardware facilities and funds, there were some defects in our research design. The sample size is not very large, and observation time is not very long. Patients and outcome assessors were not blinded to the received intervention, and thus, our data should be interpreted with caution. Finally, our study did not confirm the mechanism of DHZCP in the treatment of silicosis, although there have been previous animal results.

Although alveolar lavage can be used to treat silicosis, it is difficult to be widely used because of long duration and high cost. Compared with alveolar lavage, DHZCP is more convenient, is cheap, and has small toxic and side effects. It has definite effect on the improvement of silicosis in all aspects and is more suitable to widespread use.

## 5. Conclusion

DHZCP could improve the pulmonary function, the quality of life, and the exercise capacity of silicosis patients. This randomized controlled trial demonstrated that DHZCP is a promising pharmacological therapy for silicosis.

## Figures and Tables

**Table 1:** Baseline characteristics of the study participants.

	CG, *n* = 29		DG, *n* = 29		*p* value
	Mean (SD) or *n* (%)	CI	Mean (SD) or *n* (%)	CI	
*General information*					
Male	29 (100%)	—	29 (100%)	—	—
Female	0 (0%)	—	0 (0%)	—	—
Age, year	69.3 (8.0)	66.3 to 72.4	71.8 (9.0)	68.4 to 75.2	0.279
Height, cm	165.7 (4.1)	164.1 to 167.3	163.8 (5.1)	161.9 to 165.8	0.129
Weight, kg	63.5 (8.9)	60.1 to 66.9	61.9 ± (5.9)	59.7 to 64.2	0.427
BMI, kg/m^2^	23.1 (3.1)	21.9 to 24.3	23.1 (1.9)	22.4 to 23.8	0.933

*Complications and previous history*					
Smoke history	24 (82.8%)	—	25 (86.2%)	—	0.717
Emphysema	12 (41.4%)	—	15 (51.7%)	—	0.430
Respiratory failure	3 (10.3%)	—	3 (10.3%)	—	1.000
Tuberculosis	3 (10.3%)	—	5 (17.2%)	—	0.446
Pneumothorax	2 (6.9%)	—	4 (13.8%)	—	0.389
Bullae	3 (10.3%)	—	5 (17.2%)	—	0.446

*Pulmonary function*					
FVC % pred	66.0 (16.1)	59.8 to 72.2	62.7 (15.9)	56.4 to 69.0	0.451
DLCO % pred	63.4 (19.1)	56.0 to 70.8	62.2 (16.2)	55.8 to 68.6	0.803
*Hypoxia and exercise capacity*					
SpO_2_	88.4 (1.7)	87.8 to 89.1	88.1 (1.7)	87.5 to 88.7	0.481
6WMD	306.7 (98.6)	269.2 to344.2	306.2 (103.9)	266.7 to 345.7	0.985

*K-BILD*					
Total	56.6 (9.8)	52.8 to 60.3	56.8 (9.3)	53.2 to 60.28	0.935
Breathlessness and activities	41.1 (15.4)	35.2 to 46.9	40.3 (13.2)	35.2 to 45.3	0.823
Chest symptoms	51.3 (15.5)	45.5 to 57.2	50.6 (11.8)	46.1 to 55.1	0.833
Psychological symptoms	64.0 (6.4)	61.6 to 66.5	65.2 (7.9)	62.2 to 68.2	0.551

SD = standard deviations; *n* = number; CI = confidence interval; the sample size of FVC% and DLCO% in DHZCP group was 27 and in No DHZCP group was 28; statistical difference of smoke history was determined by the Pearson chi-square test, and other indexes were determined by the independent-samples *t*-test.

**Table 2:** Effects of different treatments.

	CG, *n* = 28		DG, *n* = 28	
	Mean (SD)	CI	Mean (SD)	CI
*Pulmonary function*				
FVC % pred	65.0 (14.7)	59.2 to 70.8	73.2 (13.8)	67.6 to 78.7
DLCO % pred	64.1 (12.2)	59.3 to 68.9	72.7 (13.5)	67.2 to 78.2

*Hypoxia and exercise capacity*				
SpO_2_	87.9 (3.0)	86.7 to 89.1	91.7 (2.3)	90.8 to 92.6
6WMD	308.6 (96.8)	271.0 to 346.1	331.1 (100.2)	292.2 to 369.9

*K-BILD*				
Total	56.6 (9.3)	53.0 to 60.1	62.6 (8.1)	59.4 to 65.7
Breathlessness and activities	40.9 (15.1)	35.1 to 46.8	48.8 (12.7)	43.9 to 53.7
Chest symptoms	51.2 (15.9)	45.0 to 57.4	61.9 (11.8)	57.3 to 66.5
Psychological symptoms	64.2 (6.6)	61.6 to 66.8	67.9 (6.5)	65.4 to 70.5

The sample size of FVC% and DLCO% in DHZCP group was 26 and in No DHZCP group was 27.

**Table 3:** Statistical differences between effects of DG and CG.

	MD	SE	CI	*p* value
*Pulmonary function*				
FVC % pred	8.15	3.92	0.28 to 16.02	0.043
DLCO % pred	8.64	3.53	1.55 to 15.73	0.018

*Hypoxia and exercise capacity*				
SpO_2_	3.79	0.72	2.34 to 5.23	<0.001
6WMD	22.50	26.33	-30.29 to 75.29	0.397

*K-BILD*				
Total	6.03	2.33	1.37 to 10.69	0.012
Breathlessness and activities	7.89	3.73	0.41 to 15.37	0.039
Chest symptoms	10.71	3.74	3.22 to 18.20	0.006
Psychological symptoms	3.74	1.75	0.23 to 7.26	0.037

Statistical differences were determined by the independent-samples *t*-test.

**Table 4:** Statistical differences after and before intervention.

	DG (after—before)				CG (after—before)			
	Mean (SD)	SE	CI	*p* value	Mean (SD)	SE	CI	*p* value
*Pulmonary function*								
FVC % pred	10.37 (9.26)	1.82	6.63 to 14.11	<0.001	−0.99 (10.32)	1.98	−5.07 to 3.10	0.624
DLCO % pred	10.51 (9.37)	1.84	6.73 to 14.29	<0.001	0.66 (19.09)	3.67	−6.89 to 8.21	0.859

*Hypoxia and exercise capacity*								
SpO_2_	3.61 (1.93)	0.37	2.86 to 4.36	<0.001	−0.50 (2.80)	0.53	−1.58 to 0.58	0.352
6WMD	39.64 (29.87)	5.65	28.06 to 51.23	<0.001	−5.54 (61.15)	11.56	−29.25 to 18.18	0.636

*K-BILD*								
Total	5.79 (3.79)	0.72	4.32 to 7.27	<0.001	−0.64 (4.36)	0.82	−2.32 to 1.05	0.447
Breathlessness and activities	8.48 (7.06)	1.33	5.75 to 11.21	<0.001	−1.04 (7.40)	1.40	−3.91 to 1.83	0.463
Chest symptoms	11.31 (7.48)	1.41	8.41 to 14.21	<0.001	−0.99 (8.69)	1.64	−4.36 to 2.38	0.551
Psychological symptoms	2.72 (3.02)	0.57	1.55 to 3.89	<0.001	−0.26 (4.64)	0.88	−2.06 to 1.55	0.773

Statistical differences were determined by the paired-samples *t*-test.

**Table 5:** The number of participants with abnormal safety outcomes.

	DG (*n* = 28)			CG (*n* = 28)		
	Baseline	Week 9	ACR (%)	Baseline	Week 9	ACR (%)
RBC	0	0	0	0	0	0
HGB	0	0	0	0	0	0
ALT	0	0	0	0	0	0
AST	0	0	0	0	0	0
BUN	0	0	0	0	0	0
SCr	0	0	0	0	0	0
Myo	6	5	0	8	6	0
CTnI	0	0	0	0	0	0
PT	0	0	0	0	0	0
INR	0	0	0	0	0	0
APTT	0	0	0	0	0	0

ACR = abnormal-changed rate (the rate of that the outcomes changed from normal to abnormal).

**Table 6:** Diarrhea in patients receiving DHZCP.

No.	Days	Mean frequency (per day)	Use antidiarrheic (days)
1	5	3	No
2	3	2	No
3	3	2	No
4	4	2	No
5	6	3	No
6	5	2	No
7	6	3	No
8	4	2	No
Mean	4.6	2.4	—

**Table 7:** The active ingredients and effects of all components of DHZCP.

Components	Active ingredients	Effects
Rheum palmatum L.	Rhein, emodin	Anti-inflammatory, antibiosis
*Scutellaria baicalensis* Georgi	Baicalein, baicalin	Antibiosis, antivirus, anti-inflammatory, immunoregulation
Glycyrrhiza uralensis Fisch	Flavonoid, triterpenoid saponin	Adrenal corticosteroid effects, antibechic, antibiosis, antivirus, immunoregulation
Persicae semen		Immunoregulation, antibechic, preventing asthma, anti-inflammatory
Semen armeniacae amarum	Amygdalin	
Paeonia lactiflora pall	Total glucosides of paeony	Anti-inflammatory, antibiosis, immunoregulation
*Rehmannia glutinosa*	Rehmannioside A	Immunoregulation, antibiosis
*Toxicodendron vernicifluum*	Urushiol, laccase	Relieve bronchospasm
*Tabanus*	Polypeptide, protide, fatty acid, polysaccharide	Anti-inflammatory, degrading fibrin and fibrinogen
Whitmania pigra Whitman		Antibiosis, anti-inflammatory
*Holotrichia diomphalia* Bates	Polypeptide, protide,	Antibiosis
*Eupolyphaga*	Total alkaloids of *Eupolyphaga*	Improve tissue tolerance to hypoxia

## Data Availability

The data that support the findings of this study are available upon request from the corresponding author. The data are not publicly available due to privacy or ethical restrictions.
